# Moving Forward after Sendai: How Countries Want to Use Science, Evidence and Technology for Disaster Risk Reduction

**DOI:** 10.1371/currents.dis.22247d6293d4109d09794890bcda1878

**Published:** 2015-05-14

**Authors:** Julie Calkins

**Affiliations:** UK Collaborative on Development Sciences, London and the University of Leeds, Department of Earth and Environment, Leeds, UK

## Abstract

**Background:** Following the 2004 Indian Ocean earthquake and tsunami event, the global community adopted the UN Hyogo Framework for Action (HFA) for Disaster Risk Reduction 2005-2015, which set out priorities to help countries achieve disaster resilience by encouraging the establishment of national platforms and strengthening disaster governance. In March 2015, UN member states adopted the successor to HFA, the Sendai Framework for Disaster Risk Reduction: 2015-2030 (SFDRR). The SFDRR recognises the cross-cutting nature of DRR policy and calls on stakeholders to help governments. Over the following months, the international science community as a stakeholder will contribute by outlining guidance, research opportunities and partnerships to help countries implement the new framework. To inform this process, this study examines government’ and national scientists’ perspectives about the needs to use science, evidence and technology to achieve disaster risk reduction (DRR) and put the words of the new framework into action.

**Methods:** This study was conducted using qualitative content analysis and quantifiable survey results. Data was collected via extraction from published statements and online survey responses. For statement content analysis, search terms were determined iteratively in a sample of statements until no new terms emerged. Additionally, 167 national scientists were recruited to participate in the online survey with a response rate of 26.3% (44/167).

**Findings:** Country priorities are clustered and clear, showing that there is a demand for greater science in DRR decision-making and solutions. The main themes highlighted by countries were promoting research and practitioner engagement; increase technology transfer mechanisms; open data; communication of usable evidence and user’s needs; education and training; and lastly, international cooperation all contributing to national capacity building. As identified, the main difficulties with existing delivery are gaps in knowledge, lack of coordination and a gap in capacity to use scientific evidence for policy-making.

**Conclusions:** Countries and organisations have identified a range of science and technology related needs, including through the preparatory and drafting process for the Sendai Framework for DRR. Across regions and development levels, countries are seeking to address the gaps they face in scientific capacities and information. It is hoped that understanding these priorities and challenges will help decision-makers and scientists in developing the implementation plan to consider how science, technology and innovation can be enabling factors for DRR. An implementation plan of action underpinned by scientific evidence has the potential to save lives, more accurately target investment, and contribute to greater resilience over the coming decades.

## Introduction

The 2004 Indian Ocean earthquake and tsunami event was historically unprecedented and exposed the vulnerability of countries and communities to natural hazards. However this event also spurred the global community to adopt the UN Hyogo Framework for Action (HFA) for Disaster Risk Reduction (DRR)^1^, designed to detail the priorities for work and practical means that are required to achieve disaster resilience. Similar to the Millennium Development Goals, the HFA ran from 2005-2015. Over the past 10 years, implementation of HFA has been hastened by external events, such as Hurricane Katrina, which serve to remind society of the consequences of poor decision-making. Nonetheless, disasters are occurring more frequently, with accelerating human and financial costs^2^ and increased impacts of events such as the 2011 Fukushima Nuclear Disaster and 2013 Typhoon Haiyan.

After extensive consultation, governments have now adopted the successor to HFA, the Sendai Framework for Disaster Risk Reduction: 2015-2030 (SFDRR), and agreed priorities for progress in mitigating, preparing for and recovering from disasters. This process culminated in the Third UN World Conference for DRR (14-18 March 2015, Sendai, Japan) where the SFDRR was agreed by 187 UN member states. The Sendai Framework^3^ sets the aim to achieve ‘the substantial reduction of disaster risk and losses in lives, livelihoods and health and in the economic, physical, social, cultural and environmental assets of persons, businesses, communities and countries’ (3 para 16)

Learning from the HFA, the Sendai Framework prioritises understanding disaster risk, strengthening collaboration at global and regional levels, and recognizing the critical role of stakeholders in enabling national action. By recognising weak institutional arrangements as drivers of risk and the need to strengthen ‘disaster risk governance’ (3 para 6), the SFDRR supports that DRR is central to development and supports the principle that decision-makers should consider disaster risk in all new investments (i.e. 3 Priority 3).

The new framework also assigns roles and responsibilities to stakeholders at the international, regional, national to local levels for achieving global disaster resilience goals. Compared with HFA, there is an enhanced role for science, technology and innovation; science is a distinct stakeholder embedded throughout the framework, with a role and specific responsibilities. While the importance of modelling and early warning systems solutions are emphasized, there is also recognition of the importance in understanding wider cultural and socio-economic processes in disasters and DRR, as well as a renewed emphasis on education and integrated approaches to training.

The SFDRR signals a clear mandate to the science, technology, and innovation community to work together with governments in developing and sharing the knowledge and solutions needed to improve the resilience of communities, save lives and reduce disaster losses. Following on from the adoption of the SFDRR, UN, governments and all stakeholders will work to translate these goals and commitments into concrete executable actions^4^. The means of implementation will call for urgent mobilization and more effective use of resources for DRR; however where to focus those efforts and resources is yet unclear.

Scientific information, technology and innovation for successful disaster risk reduction and resilience has long been recognised as essential by the international community^5^. The UN International Strategy for Disaster Reduction (UNISDR) has specified that successful disaster resilience requires scientific and technical capacities with inputs from physical, social, economic, health and engineering disciplines^6^. Recent reviews have highlighted many case study examples of science, technology and innovation being successfully used and communicated for disaster risk reduction and management5. Innovations in methods, tools and analyses have made significant progress in finding solutions, and data availability^7^.

Nonetheless the 2011 Mid-Term Review of HFA^8^ revealed a need for tailored evidence-based approaches with advocacy and investment advice, there remained little improvement in coordination of scientific entities or other steps toward meeting the demand for greater knowledge and building technical capacities^9^. Insufficient knowledge is likely to be a key barrier to the best use of science, technology and innovation for effective DRR^10^ and implementation of HFA. Weichselgartner and Kasperson^11^ have also argued that increasing disaster losses result in part from unsatisfactory interpretation of existing scientific knowledge, i.e., translating research findings into practical actions. Additionally to be useful and used information, knowledge and solutions in DRR must be produced inclusively with practitioners and local stakeholders^12^.

There is a persistent and recognised call for science to provide and share actionable knowledge with explicit links to policy and decision making^13^. In fact, countries and other major stakeholders have identified a range of science and technology related needs, throughout the preparatory and drafting process of the SFDRR. This study investigates those requirements for achieving resilience and implementation of the Sendai Framework, to inform the multidisciplinary scientific community of the opportunity for engagement and need for knowledge to action.

## Methodology

**Content Analysis** **of National Statements**

Data extraction consisted of a review and coding of statements made by countries and organisations at the first UNISDR Preparatory Committee Meeting for the post-2015 DRR Framework (July 2014), to explore the demand for science and technology. 106 statements were submitted by National Governments, intergovernmental organisations and Stakeholder Major Groups. Statements were accessed via the UNISDR World Conference for DRR website^14^. Non-English statements were translated to English using Google Translate^15^ prior to coding. The accuracy of translation via this method has been validated^16^. Nonetheless, translations of statements submitted in Arabic, Chinese, French and Spanish were additionally independently verified by native speaking research area experts to validate data extraction and context. Russian (n=2) and Portuguese (n=1) statements were not independently verified; however Portuguese has been shown to have high agreement with English translation using Google Translate^15^.

During the initial coding phase, 10 statements were read several times and key words were determined iteratively in the text, until no new terms emerged. Using validated methods^17^, primary search terms and surrounding content were extracted and categorized. Search terms: data, sci*, info*, evidence, knowledge, tech*, research, model*, edu*. During this process, the extractions were spot checked with national delegates and experts to improve trustworthiness. Coded extracts were then interpreted into data-driven themes.

**Survey**

Using the categories from the content analysis, a survey was developed. The survey consultation with national scientific bodies was intended to assess the demand for science, the usefulness and (limits to) accessibility of information from existing initiatives, and expectations for an enhanced approach in the post-2015 DRR Framework (now the SFDRR). 167 national scientists were recruited by membership through the Global Network of Science Academies (IAP) and the International Council for Science (ICSU). Invitations to participate in the online survey were sent by batch email with two evenly-spaced reminders^18^. Data analysis consisted of collecting quantitative results and coding qualitative results using the prior content analysis technique.

## Findings

At the 1st Preparatory Committee for the Sendai Framework for Disaster Risk Reduction, many Member States, Regional Groups, Intergovernmental Organisations (IGOs) and thematic Major Groups emphasized the importance of science, technology, innovation and education for effective DRR decision-making. Of 106 statements, 89 were from Governments, 8 from IGOs, and 9 from major stakeholder groups. In total, 70 out of 89 Member State and Country Group Interventions, 7 out of 8 from IGOs and 7 of 9 Major Group Interventions expressed such messages.

These statements represent the range of current views on the role of science and technology in the Sendai Framework for DRR. While this has not captured nuances of views, it is indicative of the level of support for a greater role of science and technology and the converging area of interests, priorities, and scales of needs. In addition, the survey was administered for 4 weeks over September 2014 and gathered responses from national scientists in 44 countries, including 32 Low-Income and Middle-Income Developing Countries^19^ (Response Rate: 26.3%, 44/167). Open answers to survey questions provide further context to the needs of governments and are included in the thematic analysis. An asterisk (*) denotes data collected by survey.

Figure 1 shows the individual countries from which statements and/or survey results were included. Together these datasets show that there are significant areas of agreement from diverse countries around the world on the need for science to support implementation. Five themes emerged from the statement content analysis and open survey data pertaining to the potential role of science in implementation of the post-2015 DRR Framework, the proposed focus areas and functions for science, technology and innovation (Table 1). Each theme, supported by examples is discussed, in the following section.

**Statements were included from countries in light gray; survey results were contributed by countries in dark grey. The views of striped countries are included in both statement analysis and survey results.**
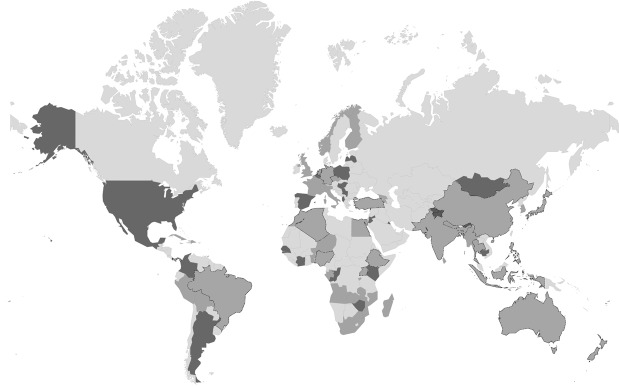

**Table 1. Themes which emerged from the statement and open survey response content analysis.** Country group statements (e.g. EU) have been counted as a unit, not as individual.

Themes	Statements	Survey
1. Increase Scientific research and practitioner engagement	28	13
2. Technology Transfer and Innovation	36	19
3. Open data access, knowledge management and sharing	31	14
4. Communication and education	28	12
5. Strengthen coordination and cooperation structures	26	10
Cross-cutting capacity building	41	23

Theme 1. Increase scientific research and practitioner engagement: This theme emerged as support for specific research areas and identification of gaps in knowledge and inclusion of practitioner. Promote scientific research into risk patterns and trends, as well as the causes and effects of disaster risk in society; and engage with the National/Sub-National research and practitioner community involved in DRR to strengthen the science-policy interface.

In addition to general calls for research and practitioner engagement, the following gaps were specified: drought and desertification (Algeria); agriculture and food production (Ethiopia, Gambia); permafrost melting (Mongolia*); risk and economic assessment (Armenia); post-disaster recovery (Australia*); disaster risk trends, patterns, and responses (Uganda); social factors (Indonesia*).

Theme 2. Technology Transfer and Innovation: The development, accessibility and transfer of technology and critically, continued technical support once applied.

This theme was characterized by responses such as: We lack equipment/ technology (hardware or software), expertise, staff so even if we are given equipment, we will not have experts to use it (Seychelles*); Mongolia lacks technology for DRR. Scientific and technological transfer is critical (Mongolia*); Nigeria is faced with a major impediment of lack of science and technology capacity (Nigeria*).

Priorities identified by countries further support this: innovation and new technology in DRR, open data and accessible research (Philippines); transfer of technology data and implementation of transferring mechanisms (Panama); transfer of technology for multi-hazards and response (Pakistan); knowledge and technology sharing and transfer (Dominican Republic); develop, through scientific and technical innovation, new methodologies on disaster risk management research and risk modelling (Peru); there is a need for technology transfer and capacity building mechanism to enhance the implementation (Zambia).

Theme 3. Open data access, knowledge management and sharing, capacity to generate good data: The collection, sharing and use of data on disasters and on DRR is essential. Member States clearly requested that information is made available and accessible at regional, national, and local levels.

For example in terms of generating data statistics: capacity building to develop loss database and accurate baselines (Cook Islands); means of generating data and information for DRR/M (Nigeria*); One of Zimbabwe’s gaps in implementing DRR is the ‘state of the data collection and analysis facilities’ (Zimbabwe*).

While countries also described priorities in terms of knowledge management, synthesis, and sharing: open data and accessible research (Philippines); strong data and knowledge management systems with seamless sharing of information and the creation of a central data and information clearing house of all existing programs, policies, best practices, scientific papers and evidence based recommendations and competencies that relate to DRR and climate change adaptation. (Trinidad and Tobago).

Theme 4. Communication and education: To support and expand information campaigns and public education on DRR leading to greater community resilience. As well as for local empowerment, to facilitate local access to data and increase collection and exchange of local and traditional knowledge in DRR

For example, countries describe gaps in: awareness raising activities and visible programmes (Pakistan*); gaps in policy briefs on various issues related to DRR/M and general education of the population (Uganda*). This is also reflected in priorities to: intensify information campaigns and public education on disaster management to prevent epidemics (Angola); increase risk education and training programs (Sri Lanka); teach DRR information in schools (Swaziland); support science communication and public education to ensure strong dialogue among stakeholders for science role in DRR/M (Indonesia*); and to promote use of indigenous and/or traditional knowledge (Brazil, Nigeria).

Theme 5**.** Strengthen coordination and cooperation structures, both transboundary and transdisciplinary: Increasing coordination of international and national partnerships, cross-disciplinary working and the benefit of existing initiatives, including to strengthen and increase the benefit of existing regional hubs/centres of excellence.

Problems that were identified included: lack of data, research and coordination between international initiatives (Egypt) There is no information sharing and networking between the related science organisations. (Jordan*);

Some countries offered suggestions to improve the linkages: strengthening international cooperation in science and technology related to disaster risk by improving existing cooperation frameworks (Algeria); Increasing benefit from existing international initiatives related to disasters (Egypt); Coordinate the existing institutions working on science and technology for DRR/M (Uganda*).

**Build capacity to recognise and use good science**

In addition to the five themes, there was a popular crosscutting issue around capacity building; specifically to increase national ability to consider evidence-based risk assessment in both investing for DRR and formulating evidence-based risk management policies.

For instance: Science and technology are pivotal to strengthen the evidence base in decision-making (Caribbean Community Countries [CARICOM]); Local institutions need to be strengthened to take a lead role in Disaster Risk Management and identify means to scale up community-based disaster risk management (Myanmar); There are gaps on every level and in every capacity. (Seychelles*)

**Quantifiable Survey Data**

The overall trend of lacking science was further revealed by answers from national scientists to quantitative survey questions, 75% (n = 33/44) see the lack of S&T as a national challenge to HFA implementation (Table 2). 73% (n = 32/44) of respondent national scientists do not think that their country currently has access to sufficient science and technical information and/or capacity to inform DRR policy and practice, 89% (n = 39/44) feel that improved coordination and stronger support for science and technology exchange would be helpful.

**Table 2. Survey results confirm the extensive lack of sufficient science, coordination and technical support to implement DRR policy.** Values in brackets specify sample from low- and middle-income developing countries.

Survey Question	Yes	No
Does the requirement for science and technology present a national challenge to implementation of DRR policy and HFA?	33 (30)	11 (2)
Does your country currently have access to sufficient science and technical information and capacity to inform DRR policy and practice?	12 (5)	32 (27)
Given your country’s requirements for science and technology and any gaps you've identified, would improved international coordination and stronger support frameworks for the exchange of science and technology be useful to achieve DRR goals?	39 (31)	5 (1)

## Discussion

Diverse countries, regional groups, and major stakeholder groups emphasized the importance of greater access to knowledge, technology, information, and education as necessary tools for effective solutions and DRR decision-making. These findings identify where attention and effort from the international community is warranted and can be most effective.

Many of the gaps and difficulties identified by countries at national level can be considered products of the fundamental barriers at the science-policy-practice interface. Previous research^11^ has suggested that the underuse of science was related to factors such as differing objectives, needs, and priorities; different institutional settings, as well as differing cultural values and understanding between knowledge generator and knowledge user. It follows that providing mechanisms for connecting solutions proposed by research and technology, with the expressed needs of users and practitioners would reduce redundancy in activities and result in more tailored, context-appropriate policies.

At the conclusion of the UNISDR Scientific and Technical Advisory Group (STAG) report on 'Using Science for Disaster Risk Reduction' at the Global Platform in May 2013^5^, the UNISDR STAG recommended that science should be key to implementing the Sendai Framework for Disaster Risk Reduction. SFDRR gives the scientific community the mandate to work with countries to develop and share the knowledge and technology needed to improve the resilience of communities, save lives and reduce disaster losses. Countries are calling on science to provide an evidence-based approach for policy development and future resilient infrastructure investment. It’s clear that the successful execution and achievement of the SFDRR outcomes, both at national and international scales, will depend on a facilitating, empowering environment that is perseverant and innovative. As an integral enabler of DRR, science and technology will need to consolidate its efforts.

**Next steps**

***Linking and building capacity. ***A recent review of international science mechanisms^4^ highlighted the need for an enhanced international partnership to improve the science-policy interface in DRR and to achieve the objectives of the Sendai Framework. The current study shows that national scientists are in agreement with this conclusion. Generally, there is no formal linkage between scientific organisations, national or thematic platforms, and users. National scientists suggest that a mandated relationship is necessary to collaborate, share tools developed across science disciplines and institutions, and improve research and technology development and use. A formal enhanced partnership should be considered to harness the existing bridging functions within organisations and support collaboration to best use these existing resources. To enable national capacity building, deliberate dialogue with scientists and those responsible for managing risk needs to be modelled and reinforced. In this way national scientific institutions, as well as decision makers, are supported from international and regional levels to 1) best work within country for implementation, 2) have access to the best tools and practice 3) sufficiently examine and tailor solutions to their specific situation.

***Communication and Steering. ***There is an absence of communication both to and from the scientific community which is causing potential contributions from science and technology to be undervalued. This was best summarized by a scientist from Indonesia: ‘Policies based on science are not always the case due to lack of communications, understanding on the importance of evidence-based strategies, and understanding on how policy makers and public perceive science’. Communication, translation, of usable evidence for policy-making needs to be a priority. Steering would help the community and countries respond to gaps in the provision of science knowledge and information in some key areas as identified by countries. Efforts should be made to review these gaps and overlaps, and to strengthen initiatives that do engage users and provide open data, as well as to raise awareness, thereby minimizing duplication of efforts.

**Limitations**

This study was a first attempt to capture and communicate the needs and priorities of countries in terms of accessing and applying science and technology for disaster risk reduction and DRR policy-making. The survey is limited by the relatively small sample size and sampling method. However, this is a broad study that included all IAP and ICSU member countries and is widely geographically representative. All participants were scientists participating as national focal points and so are likely familiar with the status of their country. A limitation of the content analysis is that these statements represent the range of current views on the role of science and technology in the post-2015 framework for DRR. Clearly this does not capture nuances of views or differences within countries, groups or disciplines; however it is indicative of the level of support for a greater role of science and technology and the differing area of interests, priorities, and scales of needs.

## Conclusions

Science hastens progress. Countries have clearly demanded greater science for DRR, having identified a range of science and technology related needs, including through the Sendai Framework for DRR. Across regions and development levels, countries are seeking to address the gaps they face in scientific capacities and information. As an essential component to the development of countries, the entire science community has the opportunity to capitalize and compound benefits from existing disciplinary and interdisciplinary scientific organisations and technical tools to best serve countries’ DRR priorities. As we move towards a more integrated and comprehensive approach to DRR, improved science and technology allows quicker coordination, communication and results. The SFDRR signals a clear mandate to the science, technology, and innovation community to work together with countries to develop and share the knowledge and technology needed to improve the resilience of communities, save lives, and reduce disaster losses. It is hoped that understanding the priorities and challenges voiced by governments and international scientists, and presented herein will help decision-makers and scientists in developing an implementation plan underpinned by scientific evidence which has the potential to save lives, livelihoods and contribute to greater resilience over the coming decades.
